# Testing an evidence-based drug abuse and violence preventive approach adapted for youth in juvenile justice diversionary settings

**DOI:** 10.1186/s40352-021-00128-8

**Published:** 2021-02-02

**Authors:** Christopher Williams, Kenneth W. Griffin, Ruchi K. Mehta, Gilbert J. Botvin

**Affiliations:** 1 National Health Promotion Associates, White Plains, New York USA; 2 State University of New York at Purchase College, Purchase, New York USA; 3 Department of Global and Community Health, George Mason University, Fairfax, Virginia USA; 4 Weill Cornell Medical College, Cornell University, New York, NY USA

**Keywords:** Youth court, Juvenile justice, Positive youth justice, Substance use and delinquency prevention, Social competence-enhancement

## Abstract

**Background:**

Universal school-based prevention programs for alcohol, tobacco, and other drug use are typically designed for all students within a particular school setting. However, it is unclear whether such broad-based programs are effective for youth at risk for substance use and violence in juvenile justice settings.

**Method:**

The present study tested the feasibility, appropriateness, and efficacy of a preventive intervention to reduce risk factors for substance use and delinquency among youth in juvenile justice diversionary settings by promoting positive youth development and building personal strengths and prosocial relationships. Participants in the study (*N* = 288) were predominantly male (69%) and in the 9th grade (14 years old) or higher (91%), received the preventive intervention, and completed confidential questionnaires at the pre-test and post-test.

**Results:**

The majority of youth who participated in the intervention rated the program topics (77.9%) and activities (72%) as appropriate for their age, would recommend it to their peers (73.6%), and would use the skills learned in the future (85.4%). Comparison of post-test adjusted means revealed that the prevention program had a significant positive impact on key knowledge, attitudes, and skills including goal-setting, stress-management, and communication skills.

**Conclusions:**

The findings indicate that an evidence-based prevention approach adapted for youth diversionary settings can be effectively implemented and well-received by participating youth, and can produce positive changes in psychosocial skills and protective factors known to prevent multiple risk behaviors among youth. Future efforts to implement substance use prevention in community juvenile justice settings may benefit from highlighting a positive youth development, skills-based approach.

## Background

Youth delinquency is a serious public health concern associated with significant societal consequences (Corso, Mercy, Simon, Finkelstein, & Miller, 2007; Mendelson, Mmari, Blum, Catalano, & Brindis, 2018; Welsh et al., 2008). In 2018, juvenile courts handled 23.5 cases for every 1000 juveniles aged 10 or older in the United States (Hockenberry & Puzzanchera, 2020). Though overall rates of juvenile justice involvement have been declining in the past decade, over 850,000 delinquency cases are disposed (i.e., a definite action taken) annually in juvenile courts (Hockenberry & Puzzanchera, 2020). Evidence shows that adolescents who become involved in the juvenile justice system are at substantial risk for social, health, and behavioral problems during their teen years and as they transition into young adulthood (Schaefer & Erickson, 2016). Arrested and court-involved youth initiate substance use earlier than other adolescents, often leading to more problematic substance use, higher recidivism, and more serious offenses in adulthood (Aizer & Doyle, 2015; Henggeler, Clingempeel, Bronidon, & Picker, 2002; Kandel & Yamaguchi, 2002; Scott, Dennis, Grella, Funk, & Lurigio, 2019). These problem behaviors have a deleterious impact on adolescent mental and behavioral health (Barnett, Perry, & Morris, 2016; Belenko et al., 2017; Coley, Sims, Dearing, & Spielvogel, 2018).

Juvenile justice approaches have traditionally emphasized individualized treatment efforts combined with discipline and punishment. However, over time, diversion from formal correctional proceedings has received more attention as an alternative approach that can reduce the negative consequences that often result from involvement in more punitive court systems (Evans, Smokowski, Barbee, Bower, & Barefoot, 2016; Wilson & Hoge, 2012). The use of diversion aligns with the mission of juvenile courts to rehabilitate youth, and diversion can minimize the potentially harmful and costly effects of a formal adjudication process (Whitehead & Lab, 1989).

In recent years, some efforts to divert youth have been guided by an asset-based conceptual model referred to as *“positive youth justice”* (PYJ; Butts, Bazemore, & Meroe, 2010; Byrne & Case, 2016). The PYJ approach encourages youth involved in the juvenile justice system to build upon their existing strengths while learning and mastering new psychosocial skills through development of prosocial relationships within their communities. These “core assets” are posited to promote successful entry into young adulthood. The PYJ approach is supported by research demonstrating that juvenile programming should rely less on correctional punishment, and more on building psychosocial maturity (Schaefer & Erickson, 2016).

There are nearly 2500 youth courts with jurisdiction over 86% of the nation’s juvenile population (Hockenberry, 2019). Youth courts are designed for young people who have committed minor offenses that would otherwise make them eligible for more punitive prosecution in juvenile court, traffic court, or a school’s disciplinary process. First time offenses typically include relatively minor acts of delinquency, truancy, and aggression (e.g., shoplifting, disorderly conduct, vandalism, traffic violations). Instead of being referred to the regular juvenile justice system, youth avoid the possibility of prosecution and a criminal record by attending youth court, where their cases are heard by a jury of their peers. These courts are administered in a variety of settings including justice, educational, and community-based programs (Howell & Lipsey, 2012; Howell, Lipsey, & Wilson, 2014). Youth courts may hold significant potential for the wide-scale dissemination of evidence-based delinquency prevention interventions (Funk et al., 2020; Hockenberry, 2019; Wilson & Hoge, 2012). However, there are few rigorous evaluations that demonstrate the efficacy of preventive intervention programs in youth court settings of any type.

Because the PYJ approach emphasizes the importance of building upon protective factors and developing prosocial ties to the community, it aligns well with a prevention science perspective that aims to promote prosocial attitudes, knowledge, skill competencies, and overall resilience (Guerra & Bradshaw, 2008; Waid & Uhrich, 2020). This prevention science perspective is exemplified by the *Life Skills Training* (LST) program, an evidence-based intervention that reduces risk for drug abuse, violence, and delinquency. Initially developed for middle school settings, LST is a universal prevention approach designed to be implemented with all students in a regular classroom (Botvin & Griffin, 2015). The program teaches personal self-management skills, social skills, and other cognitive-behavioral skills needed to reduce substance use and other problem behaviors, successfully handle the challenges of everyday lives, and increase overall resilience.

The LST program has been shown to be highly effective in a series of randomized controlled trials, including both efficacy studies and large-scale effectiveness trials. Evaluation studies have shown reductions of 50% or more in smoking, alcohol use, and marijuana use among students receiving the LST program relative to controls, as well as reductions in use of other illicit drugs and positive change in a host of risk and protective factors associated with adolescent drug abuse (reviewed in Botvin & Griffin, 2015). Several adaptations were made to the content and structure of the standard LST program to ensure that it would be feasible and appropriate to implement in diversionary settings.  

The present study was designed to test the efficacy of the LST approach adapted for youth in diversionary settings. In this study, the focus was on the impact of this approach on risk and protective factors associated with substance use and violence.

## Methods

Data in the present study were collected in order to examine the effects of the intervention on knowledge, attitudes, and skills that have been shown to be associated with substance use and violence.

### Sample

A total of 364 adolescents in juvenile justice diversionary settings agreed to participate in the study and completed the pre-test survey. Of these, 288 participants also completed the post-test survey, leading to a retention rate of 79%. The sample was predominantly white (51%) and male (69%). Youth participants reported living in urban/suburban areas (49%) and rural areas (51%). Roughly half of the participants lived in two-parent homes (52%). While nearly 90% of participants were in high school, youth ranged from 7th grade to 12th grade. Participant age ranged from 13 to 18 years old with a mean age of 14 at the pre-test assessment. Facilitators were encouraged to implement the program separately for middle and high school-age participants. See Table 1 for additional details on the demographic characteristics of the sample.

**Table 1:** Sample demographics (*N* = 288)

Demographic variable	Percentage
Gender	
Male	*69%*
Female	*31%*
Race^a^	
White	*51%*
Black or African-American	*16%*
Asian	*2%*
American Indian or Alaskan Native	*4%*
Native Hawaiian or Other Pacific Islander	*2%*
More than one race	*18%*
Ethnicity	
Hispanic	*38%*
Not Hispanic	*62%*
Living Situation	
Two-parent household	*52%*
*Mother and father*	*33%*
*Mother and stepfather*	*18%*
*Father and stepmother*	*1%*
*Mother and mother*	*1%*
Only mother	*24%*
Only father	*5%*
Some with mother/some with father	*5%*
Guardian, foster parent, or relative	*5%*
Other	*10%*
Grade	
Middle school	*11%*
*7th grade*	*4%*
*8th grade*	*7%*
High school	*89%*
*9th grade*	*18%*
*10th grade*	*35%*
*11th grade*	*22%*
*12th grade*	*15%*
Academic Performance	
Mostly As	*14%*
Mostly Bs	*39%*
Mostly Cs	*44%*
Mostly Ds	*7%*
Ds or lower	*5%*
Days Absent (in the last school year)	
None	*3%*
1–2 days	10%
3–6 days	*33%*
7–15 days	*29%*
16 or more days	*26%*

^a^Categories not mutually exclusive

Recruitment of program sites was accomplished by contacting directors primarily through online searches and through the National Association of Youth Courts website. Youth courts throughout the U.S. operate in a variety of traditional and non-traditional settings. To ensure that the present study reflects the diversity of setting type, research staff recruited 16 youth courts that held sessions within peer courts (*n* = 5), alternative school programs (*n* = 7), public schools (*n* = 3), and other community-based organizations (*n* = 1).

### Procedure

Prior to enrollment, parents were given the option to exclude their child from participating in the study by returning a signed consent form to the study investigators. Prior to the baseline data collection, participating youth were instructed that their participation in the data collection was voluntary and that their responses would be kept completely confidential. Over 95% of youth participants completed the pretest self-report assessment, which was administered via a paper survey in a separate session before the intervention began. The post-test assessment was administered via a paper survey in a session immediately after the last intervention session.

Participants were assigned a unique, confidential ID code in order to compare individual responses over time. Participating youth did not provide their name when completing the survey, and no personal information was linked to survey responses. Project staff provided instructions to participants on how to complete the survey and emphasized the confidential nature of participant responses. A Certificate of Confidentiality was obtained from the National Institutes of Health to protect the privacy of subjects by limiting the disclosure of identifiable, sensitive information. The study protocol was reviewed and approved by an authorized Institutional Review Board prior to the start of the study.

### Intervention

The intervention used in this study was an adaptation of the *Life Skills Training* program. The purpose of the adaptation was to improve the appropriateness and fit of the LST approach for youth in a diversionary setting. The intervention consisted of five small-group sessions implemented by trained facilitators. Details concerning the adaptation process as well as intervention content, facilitator training and program implementation are provided below.

#### Adaptation process

The adaptation process was guided by focus group testing and key informant interviews with juvenile justice experts, diversionary setting administrators, social workers, and youth court practitioners that were external to the present study. We asked a series of questions about the intervention feasibility, relevance, and appeal. Based on the feedback provided, several adaptations were made, including reducing the overall number of sessions to conform to the brief length of time (approximately 40 h) youth are required to attend the diversionary setting. Other adaptations included adding graphics, language, and role-playing scenarios appropriate to the target population, and adjusting the reading level of the intervention materials. Additional content was added to establish and maintain prosocial relationships within one’s community, including strategies for increasing attachments to and sense of belongingness within healthy peer groups, and family units. Content was also added to discourage offenses such as theft, vandalism, and disorderly conduct by pointing out how these behaviors negatively impact individuals and communities. Throughout the program, participants learn to view the responsibilities of young adulthood in a positive light. None of the adaptations involved changes to the underlying prevention strategy.

#### Intervention content

The adapted preventive intervention consisted of five 45-min classroom sessions. The first session, *Strengths and Goal-Setting*, teaches participants how to identify one’s strengths in six life domains: education, work, relationships, community engagement, creativity, and health and wellness. It is designed to show participants how goal-setting to build upon one’s strengths can help shape the direction of one’s life. The second session, *Decision-Making and Risk Taking*, teaches participants how to make informed decisions and introduces methods for analyzing the potential consequences associated with taking risks. The third session, *Managing Stress, Anger, and other Emotions*, teaches participants to recognize stress, anger, and other emotions and how to manage them using a variety of coping skills and relaxation techniques. The fourth session, *Communication*, introduces participants to a variety of verbal and non-verbal communication techniques and emphasizes the role of effective communication in avoiding misunderstandings. The fifth session, *Healthy Relationships*, shows participants what constitutes a healthy relationship and teaches methods to develop skills for effective social interaction. Throughout the five sessions, participants learn the knowledge and skills needed to succeed in school and in their communities and resist influences to engage in high-risk activities such as substance use, violence, and delinquency. Participants were encouraged to practice skills taught both during program implementation and outside of scheduled sessions. Table 2 outlines the session topics, goals, objectives, and key skills taught in the adapted intervention.

**Table 2:** Life Skills Training *New Directions* program scope and sequence

Session	Overall Goal	Session Objectives	Key Skills
**Strengths and Goal- Setting**	To identify one’s strengths, how to take charge of one’s life, and how goal-setting can help that.	• Understand the benefits of the Life Skills Training New Directions Program. • State the importance of understanding one’s strengths and their role in shaping the direction of one’s life. • Identify one’s strengths in six life domains: education, work, relationships, community engagement, creativity, and health and wellness. • Learn and apply a structured goal-setting model in one or more of the six life domains.	Identifying strengths; goal-setting; application of goal-setting method.
**Decision- Making and Risk Taking**	To increase ability to make informed decisions, to understand how decision-making affects risk-taking, and to analyze the potential consequences associated with taking risks.	• Recognize the role of decision-making in shaping the direction of one’s life. • Increase awareness of the effects of substances on decision-making. • Learn and apply a decision-making model in one or more of the six life domains. • Examine personal and peer group attitudes about risk. • Understand how one’s priorities affect taking risks.	Application of decision-making method; reducing risky behavior; reinforcing resistance to the use of substances.
**Managing Stress, Anger, and other Emotions**	To recognize stress, anger, and other emotions and how to manage them.	• Identify how anger and stress impact one physically, mentally, and/or emotionally. • Identify situations and events within each of the six life domains that lead to feelings of stress, anger, and other strong emotions. • Recognize how the use of substances can interfere with one’s ability to manage stress and anger and other emotions. • Learn how stress-reduction techniques can help to manage emotions and stress. • Practice applying stress-reduction techniques.	Identifying situations that provoke emotional reactions; applying relaxation and stress reduction techniques; reinforcing resistance to substances.
**Communication**	To teach effective communication.	• Define effective communication. • Learn verbal and non-verbal communication techniques. • Understand the role of effective communication in avoiding misunderstandings. • Practice sending and receiving skills (active listening; clarifying; asking questions; being specific; paraphrasing).	Practicing effective use of verbal and non-verbal communication; using active listening skills; preventing misunderstandings.
**Healthy Relationships**	To increase awareness of what constitutes a healthy relationship and develop skills for effective social interaction.	• Describe the roles and responsibilities of different types of relationships. • List the attributes of healthy relationships. • Understand warning signs of unhealthy relationships. • Distinguish between passive, assertive, and aggressive types of communication. • Learn and apply assertive refusal techniques. • Use assertive techniques to enhance prosocial behaviors and resolve conflicts.	Analyzing types of relationships; identifying healthy relationships; practicing assertiveness and conflict resolution skills.

#### Facilitator training

Before implementing the program, all facilitators completed an online training that was designed to introduce the theory, concepts, objectives, and teaching methods used in the program. The training was developed and led by a highly experienced expert trainer who monitored and moderated the discussion board, provided tailored individual coaching, and engaged in troubleshooting when problems or issues were raised. Facilitators were trained to effectively lead group discussions on various curriculum topics, and were provided with instruction on interactive teaching methods including demonstration, modeling, behavioral rehearsal, feedback and reinforcement, and how to assign behavioral “homework” for real world practice away from the youth court site. The online facilitator training reviewed the program materials. The training also provided a tutorial on using the instructor’s manual and youth court participant guide. The instructor’s manual included lesson plans and implementation guidelines for each of the curriculum sessions in order to standardize implementation and enhance fidelity. The youth participant guide included a summary of curriculum points and objectives as well as a variety of exercises and activities designed to reinforce the knowledge, attitudes and skills taught in the program. Several checkpoints were included in the online training to ensure that facilitators had achieved mastery of content material and linked activities to prosocial behavior and positive youth development.

#### Implementation

The intervention was provided to 52 cohorts of youth across 16 sites. A total of 364 youth enrolled in the study and completed the pretest assessment. Of these, 288 youth (79%) attended the sessions and completed the post-test assessment. Seventy-three percent of the participants were in cohorts of 10 or fewer (range = 2 to 18). Session duration averaged 66 min (*SD* = 23.1), with approximately 27% of sessions lasting 40 min or less; 33% lasting between 41 and 60 min; 31% lasting between 61 and 90 min, and 9% lasting more than 90 min. The sessions lasting more than 90 min typically were larger and required more time to complete the interactive activities. The majority of participants attended five class sessions (68%) or four class sessions (22%), and 10% attended three or fewer sessions. Of the 43 cohorts that provided specific class dates, nearly two-thirds (*n* = 28, or 65%) completed the five class sessions in 4 to 6 weeks, eight sites (19%) completed the program in less than 4 weeks, and 7 sites (16%) completed the program in more than 6 weeks. In order to track program implementation fidelity, facilitators completed fidelity checklist forms at the conclusion of each session and indicated the extent to which the topics and activities for each session were covered. A fidelity score was calculated representing the percentage of all program topics covered by facilitators in the class sessions.

### Measures

Participants were administered a self-report questionnaire at two time points: immediately before and after the intervention. The survey included core items assessing a series of scales measuring knowledge, attitudinal, and skills variables associated with healthy and unhealthy behaviors (including decision-making, assertiveness, stress management, and drug and alcohol refusal). Many of the measures used were derived from well-known and widely-used instruments (Botvin, Griffin, Diaz, & Ifill-Williams, 2001) and new items were developed to reflect the adapted content of the prevention program (Epstein, Botvin, Diaz, Baker, & Botvin, 1997; Macaulay, Griffin, & Botvin, 2002). At the post-test, we also asked youth participants several questions about their reactions to the program, including whether the program topics and activities were age appropriate, whether they would recommend the program to their peers, and whether they think they will use the skills in the future.

#### Demographic data

Participants provided data on gender, age, family structure, and race and ethnicity. We also assessed self-reported school attendance and academic performance.

#### Knowledge

There were 24 knowledge items on the survey, with response options of “true” and “false.” Knowledge items assessed participants’ understanding of key intervention skills including goal-setting, decision-making, stress management, relationships, communication, and assertiveness (Epstein et al., 1997; Macaulay et al., 2002). Additional knowledge items included topics on alcohol and substance abuse, self-esteem, risk-taking, and community engagement. Sample items include, “Goals should be realistic, manageable, measurable, and meaningful” (T), “When we use a firm tone of voice and speak clearly, our message is more likely to be heard and understood” (T), and “Our ability to manage our own stress does not affect our ability to make healthy decisions” (F). For data analysis purposes, all 24 knowledge items were combined into a scale with a range of 0–24, representing the number of items answered correctly, with higher scores indicating more knowledge of the intervention content.

#### Attitudes

Attitudes were measured with 13 items assessing participants’ level of agreement with statements about goal-setting, decision-making, stress management, communication, and relationships. Sample attitudinal items include: “The more you practice techniques for managing stress, the more effective they are,” “Alcohol and other drug use can help you control strong emotions,” and “Knowing your strengths can help you accomplish your goals.” The response options were on a 5-point Likert-type scales ranging from “strongly disagree” to “strongly agree.” Items were reverse-coded as necessary to construct a summary mean score with a possible range of 1–5 with higher scores indicating more desirable responses.

#### Skills

The following skills were assessed. *Goal-Setting Skills* were measured with two items asking participants how likely they would be to: (1) divide long-term goals into short-term goals in order to make them more manageable, and (2) use a goal-setting model to create and make progress toward achieving a goal. *Refusal Skills* were measured with two items asking participants how likely they would be to: (1) refuse someone who is trying to get them to do something they do not want to do, and (2) refuse someone asking them to drink alcohol. *Stress Management Skills* were measured with three items asking participants how much they agreed or disagreed with the following statements: (1) “When I have a problem or need to make an important decision, I pause, take a deep breath, and tell myself I can figure it out;” (2) “To reduce stress, I do a deep breathing exercise;” and; (3) “To reduce stress, I think of something I can say to myself that is positive and calming.” *Communication Skills* were measured with two items asking participants how much they agreed or disagreed with the following statements: (1) “When I want other people to understand me, I make sure my tone of voice, how I stand, and the expression on my face all match what I am trying to say,” and (2) “To make sure I understand others, I sometimes repeat what they say back to them, in my own words, to make sure I heard them correctly."” For each skill, items were analyzed together in a mean summary score with a possible range from 1 to 5 (Epstein et al., 1997), with higher scores represent better skills.

### Data analysis

Data were analyzed using SPSS (IBM Corp, 2019) including the statistical procedures for chi-square, t-test, analysis of variance (ANOVA), and multiple linear regression. A series of chi-square tests were computed to examine attrition rates. ANOVAs were used to examine implementation fidelity by setting type. The effectiveness of the prevention program was examined using paired t-tests, comparing the pre-test and post-test means, and multiple linear regression analyses. An effect size for the intervention was calculated using Cohen’s d statistic (Cohen, 1988). Multiple linear regression analysis also examined the impact of setting type on intervention outcomes. Analyses were conducted using listwise deletion; missingness ranged from 0% to 7% for demographic items at pre-test and 1% to 4% for the outcome items among participants who completed the pre-test and post-test.

## Results

Below we present the findings examining implementation fidelity and post-test intervention effects on knowledge, attitudes, and skills.

### Attrition analysis

Analyses were conducted to examine attrition rates from the pre-test to post-test assessments. Overall, 288 (79%) of the pre-test sample was retained at the post-test assessment. Table 3 shows the attrition rates for several demographic variables. The attrition rates between male and female respondents were not different (22.5% compared to 29.6% X^2^ (1) = 2.25, *p* = .16). There were no differences in attrition between White and non-White respondents (23.1% compared to 27.0% X^2^ (1) = .78, *p* = .41). Youth from two-parent and non-two-parent families completed the post-test at the same rate (23.0% compared to 27.0% X^2^ (1) = 0.80, *p* = .22). Youth participants who reported missing 7 or more days from school completed the post-test at a lower rate than those who missed less than 7 days (29.0% compared to 19.3% X^2^ (1) = 4.70, *p* <. 05.

**Table 3:** Attrition rates by sample demographic characteristics

	Completed Pre-test and Post-test	Did Not Complete Post-test	X^2^	*P*-value
Gender			2.25	0.16
Male	193 (77.5%)	56 (22.5%)		
Female	88 (70.4%)	37 (29.6%)		
Race			0.78	0.41
White	153 (76.9%)	46 (23.1%)		
Non-White	135 (73.0%)	50 (27.0%)		
Living Situation			0.80	0.22
Two-Parent	147 (77.0%)	44 (23.0%)		
Non Two-Parent	135 (73%)	50 (27%)		
Grade			1.27	0.31
Middle School	256 (74.2%)	89 (25.8%)		
High School	29 (82.9%)	6 (17.1%)		
Academic Performance			1.01	0.19
As and Bs	146 (77.2%)	43 (22.8%)		
Cs and Below	139 (72.8%)	52 (27.2%)		
Days Absent			4.70	0.02*
> 7 days per year	157 (71.0%)	64 (29.0%)		
< 7 days per year	130 (80.7%)	31 (19.3%)		

*p < .05

### Implementation fidelity

Overall, fidelity of implementation was high, with an average of 88.8% of topics covered (*SD* = 13.8; Range = 57% to 100%). There were significant differences in implementation fidelity by setting type, with about 81% of topics covered in youth courts in alternative school settings, compared to 93% of courts in the other setting types (*F* (2, 282) = 25.04, *p* < 0.001). Taken together, data on program implementation indicated that program attendance and implementation fidelity were generally high, although there was substantial variability across sites in terms of class size, session duration, and overall duration of the intervention.

### Youth reactions to the intervention

After the program, the majority of youth participants rated the program topics (77.9%) and activities (72%) as appropriate for their age, would recommend it to their peers (73.6%), and said that they will use the skills learned in the future (85.4%).

### Intervention effects on knowledge and attitudes

For each outcome, an effect size was calculated using Cohen’s *d* statistic (Cohen, 1988). As shown in Table 4, the preventive intervention had a significant effect on several outcomes. Participants’ knowledge of relevant psychosocial topics, as measured through 24 true/false items, significantly increased from 17.79 items (*SD* = 3.57) at pre-test to 18.27 items (*SD* = 3.71) at post-test (*p* < .05, *d* = 0.15). Respondents’ prosocial attitudes were also significantly higher at post-test (*M* = 4.00, *SD* = 0.57) than at pre-test (*M* = 3.90, *SD* = 0.54, *p* < .01, *d* = 0.21).

**Table 4:** Pre-test and post-test scores on knowledge, attitudes, and skills

***Outcome***	***Pre-Test***		***Post-Test***		***Paired Difference***	***95% CI***	***Cohen’s d***
	***MEAN***	***SD***	***MEAN***	***SD***			
***Knowledge***	17.79	3.57	18.27*	3.71	0.48	(0.11, 0.85)	0.15
***Attitudes***	3.90	0.54	4.00**	0.57	0.10	(0.05, 0.17)	0.21
***Skills***							
***Goal-Setting***	3.78	0.80	4.06***	0.74	0.28	(0.17, 0.38)	0.31
***Refusal***	3.82	0.93	3.89	0.93	0.07	(−0.06, 0.18)	–
***Stress Management***	3.51	0.95	3.84***	0.88	0.33	(0.22, 0.45)	0.35
***Communication***	3.83	0.79	4.05***	0.74	0.22	(0.11, 0.33)	0.23

*p < 0.05; **p < 0.01; ***p < 0.001

### Intervention effects on skills

As shown in Table 4, goal-setting skills increased between the pre-test (*M* = 3.78, *SD* = 0.80) and post-test (*M* = 4.06, *SD* = 0.74, *p* < .001, *d* = 0.31). Stress management skills increased from the pre-test (*M* = 3.51, *SD* = 0.95) to the post-test (*M* = 3.84, *SD* = 0.99, *p* < .001, *d* = 0.35). Communication skills increased from the pre-test (*M* = 3.83, *SD* = 0.79) to the post-test (*M* = 4.05, *SD* = 0.74, *p* < .001, *d* = 0.23).

### Role of setting type

Multiple linear regression analyses were performed to assess the impact of setting type on post-test outcomes, when controlling for the impact of participants’ pre-test scores, gender, and cohort size. As shown in Table 5, findings indicated that participants from youth court settings had significantly higher scores than participants from alternative school programs in knowledge (β = 0.21, *p* < .001), attitudes (β = 0.26, *p* < .001), stress management skills (β = 0.17, *p* < .01), communication skills (β = 0.18, *p* < .01), and refusal skills (β = 0.15, *p* < .05). Participants who attended public schools or who received the program through a behavioral health organization (BHO) setting also scored significantly higher than participants from alternative school programs on the knowledge (β = 0.12, *p* < .05), attitude (β = 0.16, *p* < .01), and skills outcomes, including communication skills (β = 0.18, *p* < .01), and refusal skills (β = 0.18, *p* < .01).

**Table 5:** Role of setting type on youth outcomes

***Outcome***	***Setting Type***						
	***Youth Court***			***Public School / *** ***Behavioral Health Organization***			***Alternative School Program(Reference)***
	***Mean***	β	95% CI	***Mean***	β	95% CI	***Mean***
***Knowledge***	19.1	0.21***	(0.11, 0.31)	19.6	0.12*	(0.02, 0.23)	16.7
***Attitudes***	4.12	0.26***	(0.15, 0.36)	4.21	0.16**	(0.05, 0.28)	3.76
***Skills***							
***Goal Setting***	4.05	0.07	(−0.06, 0.19)	4.28	0.09	(−0.04, 0.22)	3.94
***Refusal***	3.93	0.18**	(0.07, 0.30)	4.24	0.19**	(0.07, 0.30)	3.62
***Stress Management***	3.93	0.17**	(0.05, 0.29)	4.09	0.07	(−0.05, 0.19)	3.62
***Communication***	4.12	0.18**	(0.06, 0.31)	4.34	0.19**	(0.06, 0.32)	3.82

* *p* < 0.05; ** *p* < 0.01; *** *p* < 0.001

We also examined whether cohort size (i.e., the number of youth participants enrolled in the program) was associated with outcomes. These analyses showed that smaller cohorts were associated with better outcomes for knowledge (β = 0.10, *p* < .05), goal-settings skills (β = 0.16, *p* < .01), and refusal skills (β = 0.19, *p* < .01).

## Discussion

A promising strategy for extending the public health benefits of prevention science involves adapting theory-based and empirically validated intervention approaches found effective in one setting or population to new settings and/or populations. Although there have been significant advances in the field of substance abuse and delinquency prevention, much of this work has focused on universal strategies designed for all students, typically in middle school settings (Botvin & Griffin, 2015). There has been relatively little research examining the effectiveness of skills-based competency-enhancement prevention approaches when implemented outside of schools for youth at elevated risk (Knight, Maple, Shakeshaft, Shakehsaft, & Pearce, 2018). The goal of the present study was to adapt and test an effective school-based drug and violence prevention program for implementation in youth courts and other youth diversion programs.

Results indicated that exposure to the adapted intervention increased participant scores on several knowledge, attitude, and skill variables known to be associated with reduced risk behaviors among adolescents. Analysis revealed the intervention had a positive impact on goal-setting and refusal skills as well as increased self-reported stress management and communication skills from the pre-test to post-test. Additional analyses revealed that the intervention was particularly effective among participants who were exposed to the intervention in youth court settings compared to alternative school programs. Smaller cohort size was also associated with better knowledge, attitudes, and prosocial behaviors. The results indicate that the intervention was effective for a variety of key outcomes, and the findings suggest that these effects may be more likely to occur when the intervention is delivered in small groups in traditional youth courts.

Though youth courts have grown in popularity across the U.S., research examining their effectiveness has produced mixed results. Indeed, results from a recent meta-analysis revealed that teen courts were no more effective at reducing recidivism than formal processing or other diversion programs (Bouchard & Wong, 2017). Other studies have shown that youth court participation actually increases recidivism (Petrosino, Turpin-Petrosino, & Finckenaur, 2000). It may be difficult to draw conclusions about the overall effectiveness of youth courts because each court typically undertakes a unique set of activities as part of their proceedings. Most youth courts require that respondents (youth offenders) stand before a jury of their peers, who determine what penalty or disposition is appropriate given the nature and severity of the infraction. These dispositions vary by court but may include community service, writing a letter of apology to those affected, or restitution involving other activities (e.g., monetary compensation, in-kind services to the victim, indemnification). Some youth courts have added behavioral interventions to the dispositions provided, but these often have limited feasibility, such as labor- or time-intensive practices staffed by highly-trained professionals (Celinska, 2015; Lancaster, Balkin, Garcia, & Valarezo, 2011) or those that have produced limited or negative results (Klenowski, Bell, & Dodson, 2010; Pardini, 2016; Rodriguez, 2007).

Findings from the present study indicate that adding youth competency enhancement programming derived from a theoretically driven, evidence-based approach can help overcome some of the shortcomings of current youth court programming in a way that is feasible and effective. Given the large number of youth courts and their dispersion and diversity across many communities, these settings offer an important dissemination vehicle for reaching at-risk youth outside of traditional school settings where prevention programs are typically implemented. If effective prevention programs are widely disseminated, this could be an important step in transforming diversion program alternatives to the juvenile justice system.

Multi-component interventions have been demonstrated to be the most effective for youth at high risk for future delinquency (Pardini, 2016; Sales et al., 2018). The intervention used in this study (*LST New Directions),* an adapted version of the multicomponent *LST* intervention, showed initial efficacy in improving outcomes for at-risk youth. These findings have important implications for practice. Specifically, this study provides empirical support that evidence-based preventive interventions designed for traditional school settings can be effectively implemented and have a positive impact on youth participants in a diversionary setting using staff with only minimal levels of professional training. Youth courts are often staffed by community volunteers. The fact that program implementers can effectively teach the program after receiving relatively brief online training suggests that the intervention is feasible, and a variety of diversionary settings could adopt and implement this type of evidence-based intervention.

Another practical consideration is that this intervention provides initial evidence of efficacy among youth who are further along the risk continuum, suggesting that such programs are feasible for implementation across diversionary settings that serve youth who have committed a range of offenses. The intervention in this study was conducted with smaller groups than is typical with universal interventions in school settings. Smaller group sizes are likely to facilitate classroom management and enhance implementation fidelity. Findings in this study indicated that setting type was associated with youth outcomes. Implementation in alternative school settings led to lower fidelity and worse outcomes relative to the other settings. However, it is unclear whether this is the result of differences related to facilitators, the characteristics of participants, or the settings. Future research should examine the characteristics of facilitators, participants, and settings that can maximize the impact of skills building interventions for youth in juvenile justice diversionary settings.

Future research should also recognize problems with the larger juvenile justice system when developing future programming. For example, Black youth are overrepresented in the juvenile justice system (Hockenberry & Puzzanchera, 2020; National Conference for State Legislatures, 2020; Youth.gov, 2020) and are likely to receive more punitive adjudication even after controlling for prior referrals and type and severity of underlying offense (Evangelist, Ryan, Victor, Moore, & Perron, 2017). Future reform efforts are clearly needed, and greater efforts should be made to achieve equitable access to diversionary settings, and the findings from the present study suggest that positive youth justice interventions are a promising approach.

### Limitations

The current study has several limitations that should be noted. First, the study design did not include a control or comparison group, which limits our ability to examine changes in behavior over time because risk behaviors increase in this age group, even among those receiving a prevention program. A control group design would be necessary to examine whether a preventive intervention reduces the relative rate of increase for risk behaviors. Without a control group, we are unable to rule out the possibility that the observed improvements are due to the juvenile justice program rather than the preventive intervention. Improvements in social competence could also be due to normal adolescent maturation, taking the survey itself, or regression to the mean. Second, the study included a relatively small sample that is predominantly White and from two-parent households, which may not be representative of youth court participants nationally and the larger juvenile justice system (American Civil Liberties Union, 2018, Campbell et al., 2018, Hockenberry, 2019, McCord, Widom, & Crowell, 2001, Moore & Padavic, 2010). This limits the extent to which these results may be generalizable to the larger population of youth court participants. The relatively small sample size in the present study also limits the kind of analyses that could be meaningfully conducted due to constraints on statistical power. Third, other factors could have influenced who participated in the study, such as socioeconomic status, family support, and race/ethnicity.

## Conclusions

Despite these limitations, the current study has several notable strengths, including the use of a comprehensive, multi-component prevention approach targeting a diverse set of risk and protective factors, standardized measures and data collection protocols, and multivariable statistical analyses of intervention effects that adjusted for several covariates to increase analytic precision. Future research is needed with a larger sample size, greater statistical power, a control condition, and random assignment to study conditions. Finally, follow-up research is needed to determine the long-term effectiveness of the preventive intervention on recidivism and other behavioral outcomes with adolescents after release from youth court and during the transition to young adulthood.

The present findings suggest that adapting existing evidence-based interventions proven to be effective in one setting or population and applying them to another may be a time-efficient strategy for increasing the number and diversity of effective preventive interventions available to target an array of health behavior problems. In the case of this study, the results demonstrate the promise of adapting an extensively tested and empirically validated school-based preventive intervention for at-risk youth in diversionary settings. Such an approach may also hold promise for addressing the behavioral precursors of delinquency and problem behavior among other at-risk youth. Overall, this study has important implications for theory and practice, and provides a major step toward developing a feasible and effective preventive intervention for adolescents in youth courts and other diversionary settings where youth are at higher risk for substance use and violence.

## Data Availability

The dataset analyzed during the study is available from the corresponding author upon reasonable request.
